# Impact of GenX on *Zea mays*: Alterations in Morphology and Physiological Performance

**DOI:** 10.1021/acs.jafc.5c15998

**Published:** 2026-05-05

**Authors:** Andrea Sabia, Ilaria Battisti, Anna Rita Trentin, Alessandro Alboresi, Tomas Morosinotto, Antonio Masi

**Affiliations:** 1 Department of Agronomy, Food, Natural Resources, Animals, and Environment, 117130 University of Padova, Viale dell’Università 16, 35020 Legnaro, Italy; 2 Department of Biology, University of Padova, Via Ugo Bassi 58B, 35131 Padova, Italy

**Keywords:** PFAS, HFPO-DA, bioaccumulation, toxicity, maize, C4 photosynthesis

## Abstract

Hexafluoropropylene oxide dimer acid (HFPO-DA or GenX) is a highly mobile perfluoroalkyl substance (PFAS) increasingly detected in the environment, yet its impact on major crops remains poorly understood. For this reason, hydroponically grown *Zea mays* was exposed to 10, 100, and 1000 μg L^–1^ of GenX for 7 days to couple bioaccumulation and physiological performance. This compound accumulated in both roots and leaves, reaching 4.5 μg g^–1^ DW at 1000 μg L^–1^, thereby demonstrating its toxic potential. Low doses stimulated lateral root proliferation and root surface area, whereas the highest GenX level drastically reduced root diameter, biomass, and aerial growth. All treatments significantly affected photosystem quantum yields, electron transport rates, CO_2_ assimilation, and transpiration while enhancing nonphotochemical energy dissipation mechanisms and modifying the abundance of photosynthesis-related proteins. This work provides the first integrative evidence that GenX simultaneously disrupts maize hydraulics and chloroplast energy metabolism, suggesting potential yield penalties in PFAS-contaminated agroecosystems.

## Graphical abstract

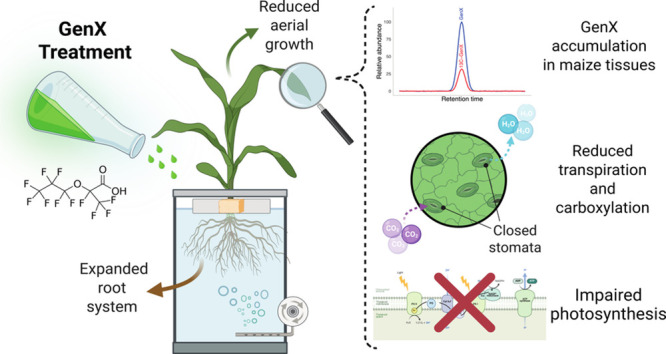

## 1 Introduction

Poly- and perfluoroalkyl substances (PFAS) are a class of more than 4,700 partially or fully fluorinated compounds, known as persistent organic pollutants (POPs). They are characterized by exceptionally strong carbon–fluorine bonds, with a dissociation energy of approximately 502 kJ mol^–1^, and by functional polar groups attached to the aliphatic chain, which confer amphiphilic properties.^1–3^ In recent years, their extensive use in industrial applications has raised significant concerns due to their exceptional chemical stability, which makes them resistant to hydrolysis, photolysis, biodegradation, and metabolism, thereby resulting in environmental persistence.^4^ Consequently, PFAS stability led to accumulation in living organisms and resulted in widespread abiotic contamination, which garnered attention due to their potential toxicity to human health and ecosystems.^5–10^

The mechanisms underlying PFAS toxicity reveal a complex interplay of oxidative stress, metabolic disruption, and interference with nutrient transport systems. For example, exposure to these pollutants in plants has been shown to induce the production of reactive oxygen species (ROS), key factors in the detrimental effects of these molecules on plant physiology, leading to cellular damage and biochemical imbalances.^11–13^ Measurements of chlorophyll fluorescence demonstrated that PFAS can alter the photosynthetic apparatus balance, confirming alterations of key genes and proteins involved in light harvesting, photochemistry, and Calvin-Benson cycle.^14^ Besides, PFAS have been found to interact with nonspecific lipid transfer proteins, as shown by *in silico* studies.^15^ Given their chemical structure, which mimics fatty acids, these compounds are capable of interfering with lipid-associated processes, potentially compromising membrane integrity and perturbing other lipid-mediated cellular functions. In addition, alterations in metabolic pathways have been documented, indicating cellular readjustments and the reallocation of resources aimed at mitigating and compensating for the incurred damage.^16–18^

In response to rising awareness of the risks associated with traditional PFAS, with the perfluorooctanoic acid (PFOA) management plan in the United States of 2006 and the Stockholm Convention on Persistent Organic Pollutants held in Geneva in 2019, the industry has sought to develop substitute compounds designed to provide similar functional benefits with presumably lower environmental and health risks. 2,3,3,3-tetrafluoro-2-(heptafluoropropoxy) propanoic acid (GenX or HFPO–DA) is one of these replacements that gained interest as a safer alternative to PFOA, with a shorter chain length and ether bond.^19^ However, evidence has established that GenX itself also presents a series of environmental and health issues.^20^ A primary concern associated with this molecule is its high solubility in water, quantified at 739 g L^–1^.^21^ This level is significantly higher by several orders of magnitude compared to certain legacy molecules, which generally display reduced sorption to soils and sediments (e.g., PFOA 9.5 g L^–1^; perfluorooctanesulfonic acid – PFOS 0.68 g L^–1^).^22^ Consequently, GenX may be less likely to be retained in the solid phase, thereby facilitating its dispersion into groundwater and surface waters, increasing its bioavailability, and enhancing its potential to cause contamination across diverse ecosystems. Recent monitoring has detected this compound in aquatic systems, especially downstream of fluorochemical manufacturing facilities in Europe, North America, and China. In several hotspots, its concentrations match or exceed those of some legacy PFAS. GenX levels in European rivers and in North Carolina have reached several hundred ng L^–1^, while surface waters in Shandong province, China, have shown up to 14 μg L^–1^ at the most impacted sites.^23–25^ Similarly, in the Veneto Region, Italy, concentrations up to 45 μg L^–1^ have been reported at the most polluted site.^26^

Despite increasing evidence of the environmental mobility and persistence of GenX, knowledge of its effects on higher plants remains limited. Current literature has primarily focused on model species or aquatic photosynthetic organisms such as *Nicotiana tabacum*, *Arabidopsis thaliana*, *Ceratophyllum demersum*, and *Chlorella pyrenoidosa*.^12,13,27^ These works elucidate that GenX has the capacity to accumulate and alter cellular homeostasis and antioxidant mechanisms. However, empirical data concerning its impact on agronomically significant crops, especially in relation to photosynthesis, remain predominantly insufficient.

Our study addresses these gaps by investigating the physiological mechanisms by which GenX impacts *Zea mays*, a globally significant C4 crop essential for food, feed, and biofuel production. The specific objectives were focused on: (i) evaluating the GenX uptake and effects on photosynthesis and gas exchange mechanisms, and (ii) investigating morphological changes and exploring the correlations between all physiological adaptations. Results from this study will provide valuable insights into the toxicity of GenX on agricultural systems and contribute to a broader understanding of how PFAS potentially penalize crop productivity and impact food security.

## 2 Material and Methods

### 2.1 Chemicals

GenX with ≥ 98% purity was purchased from SynQuest Laboratories (Alachua, FL, USA). The isotopically labeled GenX standard (^13^C_3_–GenX) was purchased from Wellington Laboratories (Guelph, ON, Canada) and added at a fixed concentration as internal standard (IS) for liquid chromatography-tandem mass spectrometry (LC-MS/MS) analysis.

### 2.2 Plant Growth and Treatment in Hydroponics Conditions

Sterilized maize seeds were germinated in darkness, and seedlings were transplanted into tall pots containing autoclaved vermiculite moistened with half-strength Hoagland’s Nutrient Solution (NS) (Supporting Information, Text S1) and maintained in a growth chamber. Seven days after sowing (DAS), plants were transplanted to polypropylene pots filled with 500 mL of aerated full-strength NS for 5 days. Full details about maize seed sterilization and germination, plantlet growth in vermiculite, and growth chamber conditions are provided in the Supporting Information (Text S1). Maize plants were equally divided into four groups (Figure S1). One group was grown in a GenX-free NS, and the other three were treated with new NS and GenX at three different concentrations, 10 μg L^–1^, 100 μg L^–1^, and 1000 μg L^–1^. These were selected to represent low and high exposure levels of GenX, respectively, based on environmental levels, as well as aligning with existing studies.^12,13,26,27^ In this context, 10 and 100 μg L^–1^ should be regarded as environmentally relevant concentrations, as they fall within the range reported in surface and groundwater collected near fluoropolymer production sites,^23–25^ whereas 1000 μg L^–1^ represents an extreme worst-case condition used to identify potential phytotoxic thresholds rather than typical field exposure. All the physiological analyses were performed 7 days after treatment (DAT). This exposure period was selected based on preliminary tests indicating that longer hydroponic cultivation under the same conditions caused nutrient and water depletion. For each condition, 6 independent biological replicates were grown (n = 6), and the findings of sections 3.2 and 3.4 are the result of two independent batches (n = 12 independent biological replicates per treatment).

### 2.3 Chlorophyll Fluorescence and P700 Analyses

The chlorophyll (Chl) content was measured every 2 days by a spectrophotometric analysis, conducted with the SPAD-502 leaf chlorophyll meter (Konica Minolta, Nieuwegein, Netherlands) on the apical central portion of the third leaf. The same leaf was assessed five times to avoid errors caused by the spatial distribution of the soil plant analysis measurements (SPAD), and the percentage variation from pretreatment (DAT0) to the last day (DAT8) was calculated. Pulse amplitude modulated (PAM) fluorescence imaging system (Fluor Cam FC 800, Photon Systems Instruments, Brno, Czech Republic) was used to assess plant stress and PSII efficiency after 40 min of darkness. The quenching analysis was carried out by measuring minimal fluorescence yield (F_0_) and maximal fluorescence yield (Fm), and the maximal photochemical efficiency (*Fv*/*Fm*). Dual-PAM-100 (Heinz Walz, Effeltrich, Germany) was used to simultaneously record photosystem I (PSI) and photosystem II (PSII) parameters under atmospheric conditions. In the induction and recovery protocol, actinic light was maintained at 553 μmol photons m^–2^ s^–1^ for 8 min, followed by 4 min of darkness to facilitate recovery analysis. To determine the light curve, the actinic light intensity was incrementally increased each minute, starting from 0.5 to 2028 μmol photons m^–2^ s^–1^, with a final 3 min period of darkness over a total duration of 20 min. The increase in light intensity did not occur at a uniform rate throughout the light curve. PSI and PSII parameters were calculated as described by Maxwell,^28^ and Klughammer et al.^29^ Photosynthetic electron transport rate (ETR) was calculated as ETR­(I) and ETR­(II) for PSI and PSII using eq 1 and eq 2, where ETR_Factor_ represents the sample absorbance, P_PS2_/P_PS1+2_ the distribution of absorbed photosynthetically active radiation (PAR) to PSII, and ϕ­(I), ϕ­(II) the effective quantum yield efficiency related to PSI and PSII, respectively:

$$\mathrm{ETR}\left(I\right)=\mathrm{PAR}\times {\mathrm{ETR}}_{\mathrm{Factor}}\times \frac{P_{PS2}}{P_{PS1}}+2\times \phi \left(I\right)$$ 1

$$\mathrm{ETR}\left(\mathrm{II}\right)=\mathrm{PAR}\times {\mathrm{ETR}}_{\mathrm{Factor}}\times \frac{P_{PS2}}{P_{PS1}}+2\times \phi \left(\mathrm{II}\right)$$ 2

### 2.4 Gas Exchange Analysis

Gas exchange analysis was performed with LI-6800 (LI-COR, Lincoln, NE, USA) using a leaf fluorometer chamber (2 cm × 3 cm) with a CO_2_ concentration of 400 μmol mol^–1^ and a flow rate of 600 μmol s^–1^. The leaf chamber’s relative humidity and temperature were kept at 65% and 25 °C, respectively. A light intensity of 1500 μmol photons m^–2^ s^–1^ was maintained for 15 min to reach steady state photosynthesis, to allow a better stomatal opening before data recording. Plants were then exposed to a decreasing light curve from 1500 μmol photons m^–2^ s^–1^ to complete darkness over 12 min. The analyses focused on the apical central section of the fourth leaf, selected due to its adequate size to completely accommodate the fluorometer chamber. Stomatal conductance (gsw), transpiration rate (E), CO_2_ assimilation rate (A), and intercellular CO_2_ concentration (Ci) were measured. All measurements were performed at similar times of the day on fully expanded and mature leaves to ensure physiological comparability among treatments. No significant variation in vapor pressure deficit or leaf temperature was observed among treatments.

### 2.5 Immunoblot Assay

Leaf disks were homogenized in 150 μL of solubilization buffer (SB) [consisting of 50 mM tris­(hydroxymethyl)­aminomethane (TRIS) (VWR, Milan, Italy) pH 6.8, 100 mM dithiothreitol (DTT) (Thermo Fisher Scientific GmbH, Bremen, Germany), 2% (w/v) sodium dodecyl sulfate (SDS) (Sigma-Aldrich, Darmstadt, Germany), and 10% (v/v) glycerol (Sigma-Aldrich, Darmstadt, Germany)] and centrifuged at 10,000 × g for 10 min. Chlorophyll was extracted using 80% acetone from aliquots of the resulting supernatant and quantitatively determined via spectrophotometric analysis (Cary Series UV–vis, Agilent Technologies, Santa Clara, CA, USA) using eq 3:^30^

$$Chlsa+b\left(\mu g{mL}^{-1}\right)=17.76\times A_{646.6}+7.34\times A_{663.6}$$ 3

Before SDS/PAGE loading, the supernatant was boiled at 100 °C for 1 min. Equal amounts of chlorophyll equivalents (2.5 μg per lane) were loaded to ensure uniform sample content. The separated proteins were transferred to nitrocellulose membranes (Millipore, Sigma-Aldrich, Darmstadt, Germany) for immunoblotting with polyclonal antibodies. Ponceau S staining of the membranes was performed to verify uniform transfer and equal protein loading across samples. Polyclonal antibodies were specific for ATP synthase gamma subunit (γATPase), Photosystem II 22 kDa protein (PsbS), Chlorophyll a-b binding protein II (LHCII), Photosystem I P700 chlorophyll A apoprotein A1 (PSAA), and Photosystem II D2 protein (D2) (custom-made). Protein detection was achieved using Horseradish Peroxidase (HRP, Agrisera, Vännäs, Sweden). Fiji software v. 2.17.0 ^31^ was used to quantify protein band intensities, which were normalized to the reference signal (Ribulose-1,5-bisphosphate carboxylase/oxygenase – RuBisCO) and expressed relative to the mean intensity of the control samples.

### 2.6 Sample Collection and Root Systems Measurement

At the end of the exposure period, after physiological measurements, the same individuals were collected for morphological measurements and LC–MS/MS quantification. This sequential approach ensured data comparability before sampling. Fresh (FW) and dry (DW) weights of root and leaf tissues were determined before and after drying at 60 °C until constant. Root morphology was evaluated with an EPSON scanning system (Epson Expression 11000 XL PRO, Nagano, Japan) after spreading the fresh root systems in a water layer on a transparent tray. Images were collected at 600-dpi resolution and analyzed with WinRHIZO software (v. 5.0, Regent Instruments, Québec City, QC, Canada).

### 2.7 GenX Quantification by LC-MS/MS

For GenX extraction, dried samples were finely ground in liquid nitrogen, and around 200 mg of powdered samples were spiked with the ^13^C_3_–GenX standard and extracted with methanol using an Accelerated Solvent Extraction (ASE) system (Dionex ASE 350, Thermo Fisher Scientific GmbH, Bremen, Germany), as previously described by Battisti et al.^10^ The accuracy and recovery of the extraction are reported in the Supporting Information (Table S2). Extracts were diluted 1:1 with H_2_O before the analysis. Mass spectrometry analysis was performed using a triple quadrupole instrument (TSQ Quantiva, Thermo Fisher Scientific GmbH, Bremen, Germany), operated in selected reaction monitoring (SRM) mode, interfaced with an ultrahigh performance liquid chromatography (Ultimate 3000 UHPLC, Dionex, Thermo Fisher Scientific GmbH, Bremen, Germany). Details on the analytical method are provided in the Supporting Information (Text S2). Raw files were processed with Skyline MS software v. 21.2.0.425.^32^ The linearity of the matrix-matched standard calibration curve was assessed between 0 and 40 μg L^–1^; the limit of detection (LOD) and the limit of quantification (LOQ) values were calculated as the analyte peak with a signal-to-noise ratio of 3 and 10, respectively, and are reported in the Supporting Information (Table S2). Glassware and all materials potentially containing fluorinated polymers were avoided to prevent background contamination.

### 2.8 Statistical Analysis

Statistical analysis was performed with GraphPad Prism software v. 9.5.0.^33^ Root morphology and gas exchange data were analyzed by one-way ANOVA (Analysis of Variance) with Dunnett test for multiple comparisons. Biomass data were statistically assessed through one-way ANOVA and the Holm-Šídák test. GenX bioaccumulation data were tested with the Kruskal–Wallis test and Dunn test for multiple comparisons. Photosynthesis-related data were tested by two-way ANOVA with Dunnett multiple comparison test, and the Geisser-Greenhouse correction was used. Prior to the analysis, data were tested for normality and homoscedasticity, potential outliers were tested by the ROUT’s test, and removed from the data set before further statistical analyses. The number of data points excluded as outliers was recorded for each data set. Postremoval, normality, and homoscedasticity assumptions were reverified to ensure the robustness of the analyses. The data are considered statistically valid according to the following pattern: * *p* < 0.05, ** *p* < 0.01, *** *p* < 0.001, **** *p* < 0.0001.

## 3 Results and Discussion

### 3.1 GenX Bioaccumulates in *Zea mays* Tissues

Exploring the translocation and distribution of GenX in maize represents a key step toward clarifying its environmental fate in crops. Our findings indicate that maize efficiently takes up GenX, accumulating it in both roots and leaves in a dose-dependent manner (Figure 1 and Table S4). At the minimal tested concentration of 10 μg L^–1^, GenX was already detectable in aerial parts, where it reaches a concentration of approximately 150 ng g^–1^ DW, documenting its high bioavailability and translocation potential through the vascular system (Figure 1A). This implies that even low environmental contamination may suffice to ensure its systemic distribution across plant tissues, generating risks for the food industry and human health. When the concentration is raised to 100 μg L^–1^, the detected level increases to roughly 180 ng g^–1^ DW, and at 1000 μg L^–1^, it rises to about 4600 ng g^–1^ DW, with statistical significance. A comparable trend was evident in roots (Figure 1B).

**Figure 1:**
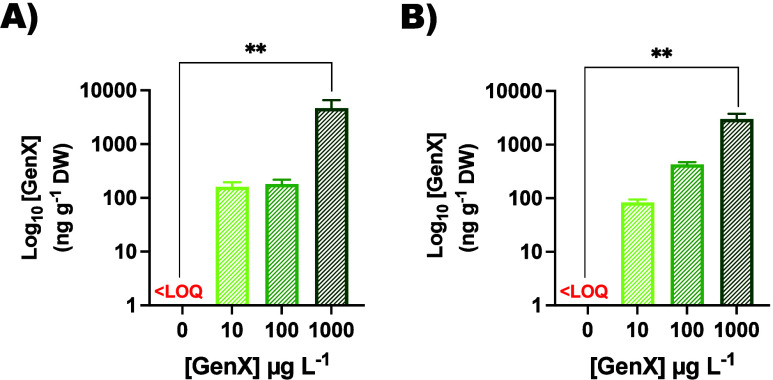
GenX content was determined in (A) leaves and (B) roots of maize plants (ng g^–1^ DW). Statistical significance is indicated by black asterisks (*p < 0.05, **p < 0.01, ***p < 0.001, ****p < 0.0001, one-way ANOVA). Data are represented on a log_10_ scale by barplots: Control in red (0 μg L^–1^) and plants exposed to GenX (10, 100, and 1000 μg L^–1^) are colored in shades of green.

Yet, as observed for xenobiotics in plants, GenX tissue levels did not scale linearly with external exposure but instead followed a nonlinear pattern consistent with complex dose–response behavior. Within a plant-pharmacology framework, these nonproportional kinetics are expected when physicochemical constraints on root permeability and long-distance transport cause uptake and translocation to plateau or shift with dose rather than scale linearly. Such patterns are better described by logistic-kinetic than simple diffusion models.^34–36^ Published studies further demonstrate that GenX bioaccumulation exhibits pronounced species dependence, with certain plant species attaining higher tissue concentrations than PFOA or perfluorobutanoic acid (PFBA), whereas others maintain comparatively lower levels.^13,37,38^ An additional methodological explanation can be attributed to GenX dimerization, which is known to occur upon electrospray ionization in the mass spectrometer source.^39,40^ This artifact may cause an underestimation of the monomer by LC-MS/MS analysis, thereby affecting the quantitative accuracy. From a biological perspective, GenX was reported to associate into small micelles at concentrations above 150 mM in aqueous solution,^41^ suggesting nonlinear uptake dynamics, thus making predictions of tissue accumulation largely unreliable. Its subcellular localization and binding affinity, indeed, remain largely unknown, limiting our capacity to fully comprehend how GenX disrupts organelle function and cellular metabolism. To minimize this bias, we systematically optimized culture conditions, sample preparation, chromatographic separation, ionization settings, and the concentrations reported here represent the most accurate quantification achievable under current LC-MS/MS methodologies.

### 3.2 Root Architectural Remodeling and Growth Responses under GenX Exposure

To investigate whether GenX exposure altered root architecture, root systems were scanned and analyzed with WinRHIZO software to quantify key morphological traits, including surface area, volume, diameter, and root tips (Figure 2 and Table S5). Notably, plants treated with 10 and 100 μg L^–1^ exhibited greater lateral root development, whereas those exposed to 1000 μg L^–1^ showed a reduction in overall structural organization (Figure 2A). Surface area of plants treated with 10, 100, and 1000 μg L^–1^ of GenX had an average percentage increase of 38.8%, 56.1%, and 19.2%, respectively, compared to control plants (Figure 2B). Similarly, root volume rose by 32.6%, 41.6%, and 17.6% (Figure 2C). Root diameter and tip number followed a similar trend, with marked stimulation at the lowest GenX level, but a pronounced decline at 1000 μg L^–1^ (Figure 2D,E). This biphasic or hormetic response is frequently observed in plants exposed to low levels of environmental contaminants, such as heavy metals or PFAS, and is generally interpreted as an adaptive strategy to optimize water and nutrient uptake while minimizing direct contact with the pollutant.^42,43^ On the contrary, excessive polluting levels may exceed physiological tolerance limits, likely due to cell toxicity or hormonal imbalance.^15,43,44^ Several investigations conducted on plants show that PFAS cause significant changes in both primary and secondary metabolism.^17,18,45^ Indeed, proteomic analyses in maize roots revealed adjustments in fatty acid and amino acid metabolism,^15^ suggesting that these compounds interfere with fundamental biochemical pathways governing growth and stress responses. In this context, the morphological effects observed under GenX exposure are consistent with those reported for legacy PFAS, supporting the idea that despite its structural differences, GenX similarly perturbs root metabolic homeostasis.

**Figure 2:**
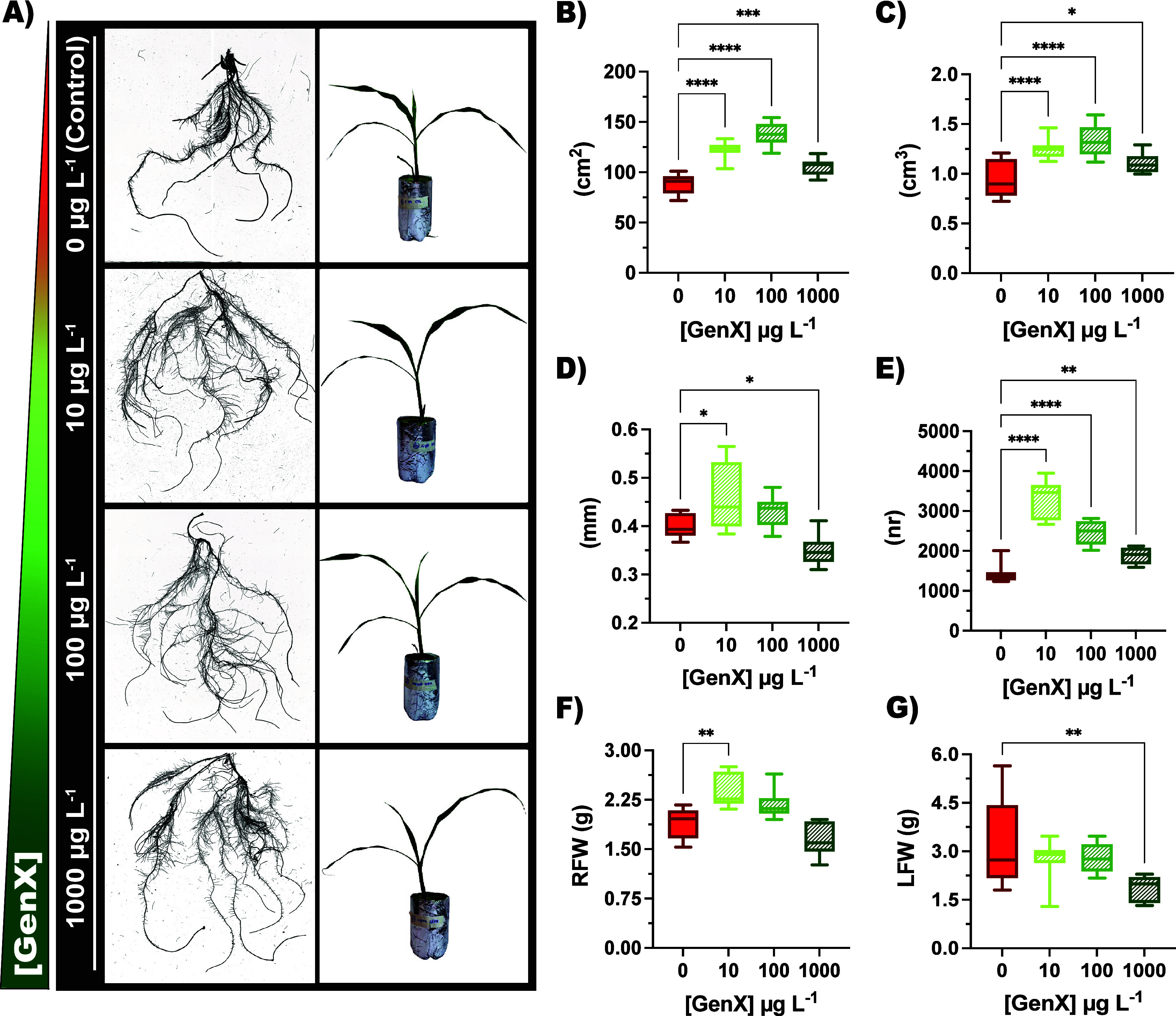
Morphological analysis is represented as follows: (A) Representative images of root systems and plant images acquisition exposed to different doses of GenX, (B) root surface area (cm^2^), (C) root volume (cm^3^), (D) root diameter (mm), (E) root tips (nr), (F) RFW (g), (G) LFW (g). Statistical significance is indicated by black asterisks (* *p* < 0.05, **p < 0.01, ***p < 0.001, ****p < 0.0001, one-way ANOVA). Data are represented by boxplots: Control in red (0 μg L^–1^) and plants exposed to GenX (10, 100, and 1000 μg L^–1^) are colored in shades of green.

Root Fresh Weight (RFW) mirrored the morphological pattern previously observed, increasing by 25.2% and 14.5% at 10 and 100 μg L^–1^, respectively, compared to control plants (Figure 2F and Table S5). Besides, at the highest concentration of 1000 μg L^–1^, RFW decreased by −13.6%, consistent with the reduction in average root diameter highlighted in Figure 2D. Similarly, Leaves Fresh Weight (LFW) exhibited a concentration-dependent trend across treatments, decreasing from control by −15.4%, −14.7%, and −43.4% (Figure 2G and Table S5), suggesting an imbalance in resource allocation between below- and aboveground organs. Such effects, as previously reported for PFAS, are the results of interferences with central pathways of carbon, lipid, and amino acid metabolism and consequently restrictions in overall plant growth capacity (Figure 2A). The marked inhibition of plants growth, especially at the environmentally relevant concentration of 10 μg L^–1^, points also to systemic physiological stress, involving impairments in photosynthetic efficiency and gas exchange, aspects further addressed in the following sections.

### 3.3 Stomatal Dysregulations Induced by GenX Stress

By measuring NS consumption, transpiration rate, net CO_2_ assimilation, and intercellular CO_2_ concentration, we gain insight into how stressed plants regulate water loss and carbon fixation (Figure 3 and Table S5). Figure 3A illustrates a negative trend in the uptake of NS across all GenX concentrations at 8 DAT. The decline was evident at 10 and 100 μg L^–1^ GenX, while the most pronounced reduction occurred at 1000 μg L^–1^, where solution consumption dropped to its minimum. Besides, the findings in Figure 2 add complexity to these results. Although an expanded root system may be perceived as an adaptive stress response aimed at enhancing water and nutrient acquisition, its functional effectiveness seems altered. Figure 3B illustrates that the transpiration rate exhibits a noticeable decline at all GenX levels: reductions of approximately −20.9% at 10 μg L^–1^, −18.4% at 100 μg L^–1^, and −29.8% at 1000 μg L^–1^ relative to the control were indeed observed. This consistent decline suggests impaired stomatal conductance, possibly driven by altered leaf water potential or abscisic acid-mediated closure mechanisms triggered by root stress signals.^15^ The concurrent limits in the rate of CO_2_ assimilation and intercellular CO_2_ concentration (Figure 3C,D) underscore the influence of GenX on stomata opening. A reduction in the assimilation rate, from 24.8 μmol m^–2^ s^–1^ in control plants to 22.0 and 21.7 μmol m^–2^ s^–1^ at minimal and maximal GenX concentrations, respectively, suggests a dysregulation in the efficiency of the photosynthetic apparatus and metabolic balance. This decline in carbon assimilation leads to a subsequent decrease in intercellular CO_2_, evidenced by the reduction from 141.7 μmol mol^–1^ in the control group to a minimum of 62.5 μmol mol^–1^ in 10 μg L^–1^ treated samples. Notably, clear impairments were already evident at 10 μg L^–1^, indicating that key processes related to stomatal conductance and carbon fixation are affected at environmentally realistic concentrations. Mechanistically, GenX, as many other legacy compounds, probably interferes with root membrane permeability or aquaporin-mediated transport, due to its amphiphilic nature, altering lipid bilayers and protein activity.^46^ Indeed, the observed decreases in transpiration rate and net CO_2_ assimilation suggest that both hydraulic conductivity and stomatal function are compromised. Such effects are also consistent with PFAS accumulation within the root cortex and vascular bundle, previously detected by desorption electrospray ionization mass spectrometry (DESI-MS) and transmission electron microscopy equipped with energy-dispersive spectroscopy (TEM-EDS) imaging in hydroponically grown plants, which revealed PFAS association with xylem parenchyma and endodermal barriers.^47^ Moreover, PFAS-induced xylem transport limitations were reported in *Salix triandra*, which stems exposed to a mixture of 11 legacy compounds exhibited increased vulnerability to cavitation and embolism, leading to impaired water transport and reduced gas-exchange capacity.^48^ The consequent decline in intercellular CO_2_ concentration limits substrate availability for RuBisCO carboxylation, reinforcing a feedback loop of reduced photosynthetic efficiency and enhanced photorespiration.

**Figure 3:**
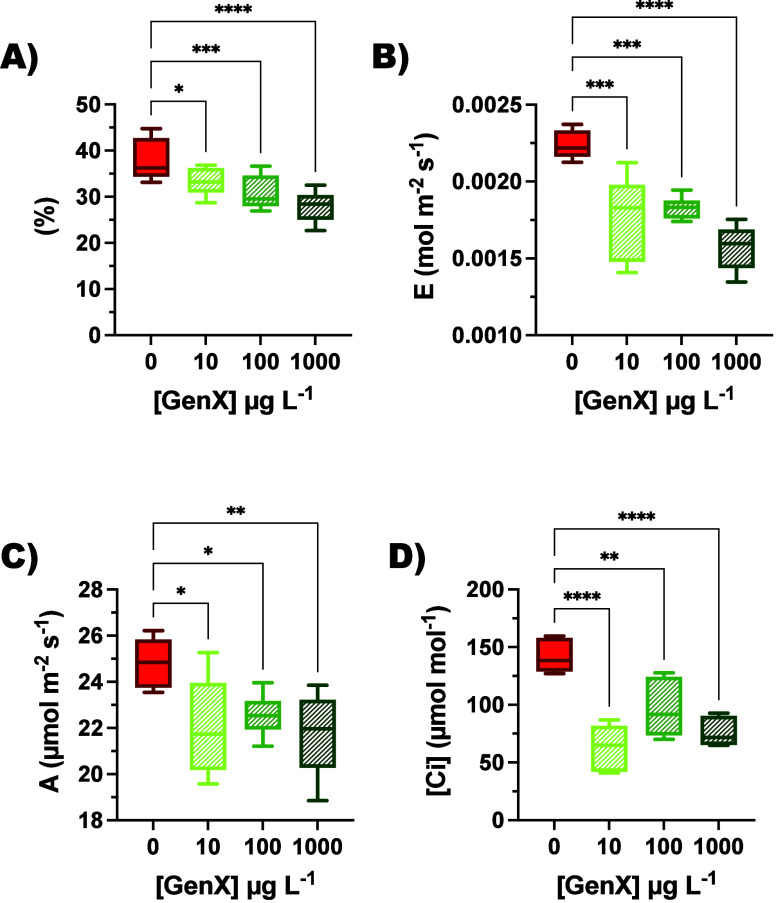
Gas exchange parameters were monitored and are represented as follows: (A) NS consumption (%), (B) transpiration rate (mol m^–2^ s^–1^), (C) CO_2_ assimilation rate (μmol m^–2^ s^–1^), (D) intercellular CO_2_ (μmol mol^–1^). Data are represented as values at a single light intensity (1000 μmol photons m^–2^ s^–1^) of a decreasing light kinetics. For full light kinetics, see Supporting Information (Figure S2). Statistical significance is indicated by black asterisks (*p < 0.05, **p < 0.01, ***p < 0.001, ****p < 0.0001, one-way ANOVA). Data are represented by boxplots: Control in red (0 μg L^–1^) and plants exposed to GenX (10, 100, and 1000 μg L^–1^) are colored in shades of green.

### 3.4 Impact on Photosynthetic Properties

Exposure to GenX caused effects on maize plants, with clear alterations in growth dynamics, root morphology, and leaf physiology. Among the various functional traits analyzed, photosynthesis emerged as a particularly sensitive target, consistently displaying signs of stress and impaired performance. Considering the centrality of photosynthetic processes in sustaining crop productivity and the fact that existing literature on GenX phytotoxicity remains scarce compared to legacy PFAS, a deeper examination of its impact on the photosynthetic apparatus is essential.^28^ Initially, the chlorophyll content was assessed by the SPAD analysis, which serves as a swift, noninvasive measure for leaf chlorophyll levels and facilitates the evaluation of pigment’s temporal variations. Following this, the photosynthetic performance was investigated through fluorescence imaging, enabling the calculation of *Fv/Fm*, combined with the recording of response curves under varying actinic light intensities, to examine energy redistribution between photosystems and the equilibrium of photoprotective mechanisms influenced by this abiotic stress. Besides, the immunoblot results provided additional insights into the molecular response differences between control and treated samples.

GenX exposure led to a significant impact on the photosynthetic apparatus with an increase in SPAD-derived chlorophyll units quantified at DAT0 and DAT8 of the treatment. It showed a 2.4% growth in control plants, while plants treated with 10, 100, and 1000 μg L^–1^ of GenX showed larger rises of 15.4%, 14.4%, and 8.4%, respectively (Figure 4A and Table S5). This pattern is consistent with the increase in total Chl (a + b) across all treatments (Table S5), and with other studies reporting increased pigment production at low PFAS levels.^49,50^ In addition, major antenna complexes, such as LHCII, presented higher accumulation at all GenX concentrations compared to control plants (Figure 5D and Table S7). In these scenarios, increased pigment content may represent a compensatory response to the stress driven by upregulation of genes involved in the synthesis of photosynthesis-antenna proteins.^27,51^ Despite chlorophyll content increases, the functional state of the photosynthetic apparatus was negatively impacted, showing a significant reduction in PSII efficiency (Figure 4B and Table S5). Under optimal conditions, *Fv/Fm* typically approaches a value of 0.79–0.80 in most maize hybrids, and its reduction is often observed under stress conditions.^52^ Importantly, decreases in *Fv/Fm* were not restricted to the 1000 μg L^–1^ treatment but were already measurable at 10 μg L^–1^, indicating that the photosynthetic machinery is highly sensitive to GenX even at environmentally relevant doses. In line with this decline in PSII performance, treated plants demonstrated reduced ϕ­(II) and ϕ­(I) compared to the control (Figure 4C,D and Table S6). Notably, following the initial transition from dark to light, ϕ­(I) of samples exposed to 100 and 1000 μg L^–1^ of GenX exhibited minimal initial values, showing a difference of about 0.1 (Control = ∼ 0.72; Treated lines = ∼ 0.62) (Figure 4D), implying an intrinsically reduced ability to employ absorbed light energy for photochemistry. Then, upon exposure to a stronger light (>300 μmol photons m^–2^ s^–1^), both PS yields of all treated lines decreased, showing a gap as high as 0.08 with respect to the control. Besides, the 1000 μg L^–1^ group exhibited a slower and incomplete recovery of ϕ­(II) (Figure 4C), suggesting a persistent photoinhibition or impaired repair of the D1 and D2 protein within PSII. These conditions were reported in *Sorghum bicolor* and *Chlorella pyrenoidosa*, where reduced photochemical efficiency and downregulation of energy transduction genes have been highlighted.^27,53^

**Figure 4:**
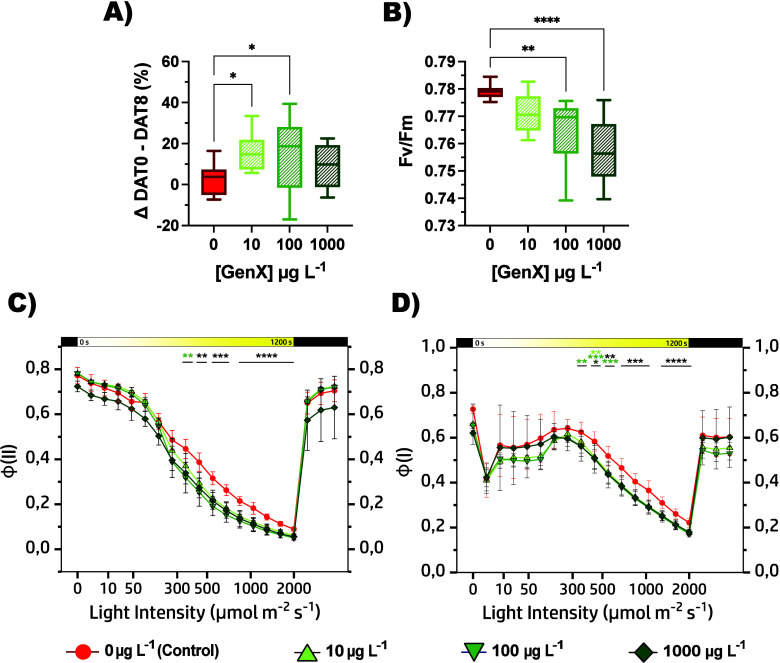
(A) Chlorophyll accumulation represented in SPAD Units (Δ% T0–T8), and (B) *Fv/Fm* are shown. Photosynthetic parameters were also monitored during light exposure and are represented as follows: ϕ­(II) (C), ϕ­(I) (D). At time 0, after 40 min of dark adaptation (first black bar), plants were exposed to light (from 0 μmol photons m^–2^ s^–1^ to 2028 μmol photons m^–2^ s^–1^; yellow gradient bar) for 20 min, followed by a rapid recovery in the dark for 2 min (second black bar). Data represent mean ± standard deviation, with statistical significance indicated by black asterisks (groups ≥ 2) and green asterisks (single group) (*p < 0.05, **p < 0.01, ***p < 0.001, ****p < 0.0001, two-way ANOVA). Data are represented by boxplots and kinetics: Control in red (0 μg L^–1^) and plants exposed to GenX (10, 100, and 1000 μg L^–1^) are colored in shades of green.

**Figure 5:**
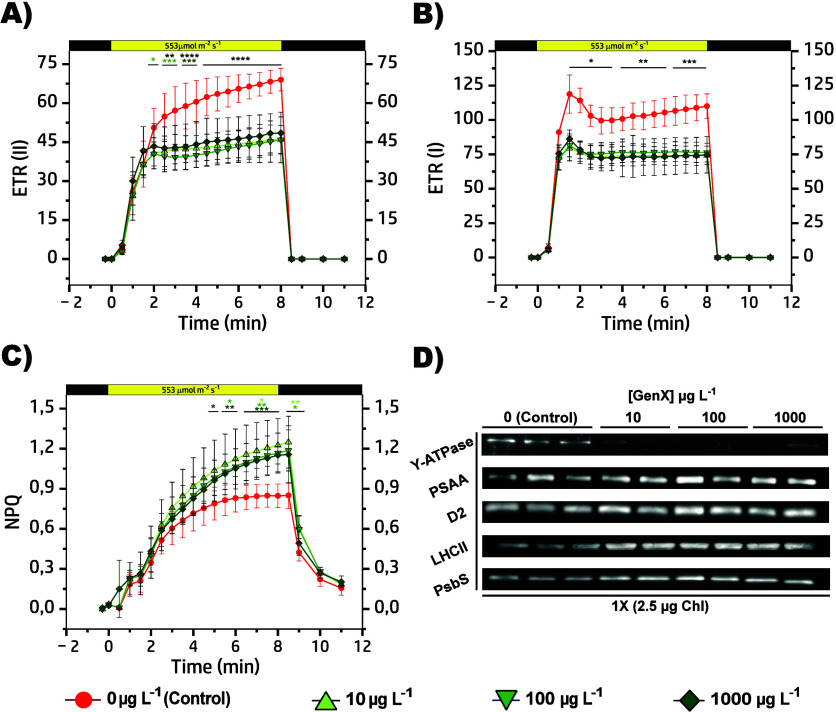
ETR (II) (A), and ETR­(I) (B), as well as NPQ (C), were monitored during light exposure. At time 0, after 40 min of dark adaptation (first black bar), plants were exposed to a nonsaturating actinic light of 553 μmol photons m^–2^ s^–1^ (yellow bars) for 8 min, followed by a recovery in the dark for 4 min (second black bar). Data represent mean ± standard deviation, with statistical significance indicated by black asterisks (groups ≥ 2) and green asterisks (single group) (*p < 0.05, **p < 0.01, ***p < 0.001, ****p < 0.0001, two-way ANOVA). Data are shown in red for Control (0 μg L^–1^), whereas plants exposed to GenX (10, 100, and 1000 μg L^–1^) are colored in shades of green. Immunoblot analysis (D) was performed to detect photosynthesis-related proteins in both control and treated lines (10, 100, and 1000 μg L^–1^). The proteins analyzed included: γ-ATPase, PSAA, D2, LHCII, and PsbS. The detection was carried out using 2.5 μg of chlorophyll, and each lane describes an independent biological replicate.

We also examined the dynamic changes in ETR­(II) and ETR­(I), and nonphotochemical quenching (NPQ). A marked limitation was observed in both ETR for GenX-treated samples (Figure 5A,B and Table S6). After dark adaptation, indeed, ETR­(II) rapidly increased within the first 2 min of light exposure, reaching a maximal value of ∼45, even at 10 μg L^–1^, which remained constant until the light was switched off. This plateau often correlates with an early saturation of PSII activity, reflecting restricted electron flow or damage to the oxygen-evolving complex. By contrast, the control plants continued to increase their value up to ∼70 after 8 min, indicating a more efficient photochemical capacity and sustained redox turnover. A similar pattern was evident for ETR­(I), where a minor initial gap between control and treated samples progressively widened, reaching a difference of ∼35 at the end of the light phase. Several studies evidenced PFAS-induced alterations in chloroplasts and thylakoid membranes architecture, as observed in *Eichhornia crassipes*, *Cyperus alternifolius*, and *Ipomoea aquatica* exposed to PFOS or 14:2 Cl-PFAES ion (F-53B).^50,54^ These plants exhibited disrupted membrane stability, enlarged intercellular spaces, and mitochondrial damage, hallmarks that could similarly apply to GenX and easily explain the previous results. Crucially, we also observed a strong dose-dependent reduction in ATP synthase γ-subunit expression with almost undetectable protein bands (Figure 5D and Table S7). This subunit is essential for the catalytic activity of the chloroplast ATP synthase complex, which couples proton translocation across the thylakoid membrane for ATP synthesis. Hence, the ATP required to drive the Calvin-Benson cycle and other anabolic processes becomes limited, further enhancing plants’ physiological stress, as previously observed under gas exchange limitations (Figure 3). Indeed, several transcriptomic, metabolomic, and proteomic studies showed that PFAS exposure perturbs key pathways in carbon metabolism, TCA cycle function, and ATP regeneration.^15,17,18,27^

In parallel, all treated plants showed an enhanced NPQ response that continued to rise throughout light exposure, ultimately reaching values significantly higher than the control (∼1.2), yet still not representing the maximal level (Figure 5C and Table S6). Besides, increases in PsbS protein levels (Figure 5D and Table S7) suggest the activation of thermal energy dissipation as a dominant photoprotective mechanism.^11,55^ This phenomenon is likely a response to reduce overexcitation and ROS formation, a process that has been extensively documented in the presence of fluorinated molecules.^11–14,45^ Nevertheless, this strategy appears insufficient to fully compensate for the stress induced by GenX. The enlargement of the antenna complex enhances light-harvesting capacity but, under conditions of constrained photochemical efficiency, as evidenced by reduced *Fv/Fm*, ϕ­(II), and ETR, results in excessive excitation pressure on PSII. This, in turn, promotes increased energy dissipation via NPQ and likely favors the formation of ROS, accelerating photoinhibition. In the control group, the larger efficiency to utilize the light energy provided for photochemistry reduces the necessity for dissipation of excess energy through nonphotochemical pathways (Figure 5C). Future research should aim to integrate omics methodologies to ascertain whether the toxicity of GenX predominantly manifests as an obstruction of energy pathways, a disruption of structural integrity, or both.

Overall, although in hydroponics, the detrimental effects of GenX that we observed on the physiology of maize plants were clearly detectable after 7 days of exposure. It may be hypothesized that, under prolonged conditions, the pronounced inhibitory effects are likely to lead to reduced plant productivity and, ultimately, to adversely affect the food market.

In soils, the mobility and bioavailability of PFAS are governed by multiple interacting physicochemical and biological factors. Sorption to soil organic matter can enhance PFAS retention, particularly for long-chain compounds such as PFOS and PFOA. In contrast, elevated ionic strength in the soil solution can attenuate PFAS adsorption to solid surfaces, thereby increasing their mobility.^56^ Multivalent cations, such as Ca^2+^, may function as bridging ions between PFAS molecules and mineral or organic surfaces, while higher ionic strength can also enhance the solubility of short-chain PFAS. In addition, rhizosphere-associated microbial communities, particularly those belonging to the families *Sphingomonadaceae* and *Rhizobiaceae*, are known to interact with PFAS.^44^ The combined influence of these processes ultimately controls plant uptake: increased soil organic matter generally decreases PFAS uptake by enhancing adsorptive retention, whereas higher ionic strength promotes the desorption and mobilization of adsorbed PFAS, leading to greater bioavailability for root absorption. For these reasons, transitioning from controlled hydroponic to soil-based systems would be fundamental to assess whether the physiological impairments observed here scale up to field conditions, ultimately determining the real-world impact of GenX contamination on crop performance and food safety.^57,58^

## Supplementary Material



## Notes

Maize seeds germination and plant growth (Text S1); GenX quantification by LC-MS/MS (Text S2); composition of the Hoagland nutrient solution used in the study (Table S1); recovery (%) of GenX extraction by ASE, coefficient of variation, LOD, LOQ, and correlation coefficients of matrix-matched standard calibration curves (Table S2); LC-MS/MS optimized parameters for GenX detection (Table S3); accumulation of GenX in maize tissues (Table S4); quantitative characterization of maize root morphology traits, plant biomass, photosynthetic performance, transpiration, and carboxylation rates (Table S5); variation in photosynthetic performance parameters (Table S6); relative variation (%) in photosynthetic protein abundance (Table S7); plant image acquisition at 12 DAS (Figure S1); gas exchange kinetics for transpiration, stomatal conductance, CO_2_ assimilation, and intercellular CO_2_ (Figure S2)­(PDF)

## a

Department of Biomedical Sciences, University of Padova, Via Ugo Bassi 58B, 35131 Padova, Italy

## Abbreviations

ANOVA: Analysis of variance
ASE: Accelerated solvent extraction
CID: Collision-induced dissociation
Chl: Chlorophyll
D2: Photosystem II D2 protein
DAS: Days after sowing
DAT: Days after treatment
DESI-MS: Desorption electrospray ionization mass spectrometry
DTT: Dithiothreitol
DW: Dry weight
ETR: Electron transport rate
ETR­(I): Electron Transport Rate of Photosystem I
ETR­(II): Electron Transport Rate of Photosystem II
F0: Minimal fluorescence yield
Fm: Maximal fluorescence yield
*Fv*/*Fm*: Maximum Quantum Efficiency of PSII
FW: Fresh weight
GenX/HFPO–DA: Hexafluoropropylene oxide dimer acid
HRP: Horseradish peroxidase
IS: Internal standard
LC-MS/MS: Liquid chromatography tandem mass spectrometry
LFW: Leaf fresh weight
LHCII: Light-Harvesting Complex II
LOD: Limit of detection
LOQ: Limit of quantification
NPQ: Non-photochemical quenching
NS: Nutrient solution
PAM: Pulse-amplitude modulated fluorescence
PAR: Photosynthetically active radiation
PFAS: Poly- and perfluoroalkyl substances
PFBA: Perfluorobutanoic acid
PFOA: Perfluorooctanoic acid
PFOS: Perfluorooctanesulfonic acid
POP: Persistent organic pollutant
PS: Photosystem
PSAA: Photosystem I P700 chlorophyll a apoprotein A1
PsbS: Photosystem II 22 kDa protein
PSI: Photosystem I
PSII: Photosystem II
ROS: Reactive oxygen species
RFW: Root fresh weight
RuBisCO: Ribulose-1,5-bisphosphate carboxylase/oxygenase
SB: Solubilization buffer
SDS: Sodium dodecyl sulfate
SPAD: Soil plant analysis development
SRM: Selected reaction monitoring
TEM-EDS: Transmission electron microscopy equipped with energy-dispersive spectroscopy
TRIS: Tris­(hydroxymethyl)­aminomethane
UHPLC: Ultra-high performance liquid chromatography
γATPase: ATP synthase gamma subunit
μg L–1: Micrograms per liter
