# Supplementary material for: Impact of GenX on Zea mays: Alterations in Morphology and Physiological Performance
Source: J Agric Food Chem. 2026 May 5;74(18):14286–96. doi: 10.1021/acs.jafc.5c15998 (PMC13178074; doi:10.1021/acs.jafc.5c15998)
Supplement: Supplementary file 1 [file jf5c15998_si_001.pdf]

Supporting Information

Impact of GenX on *Zea mays*: Alterations in Morphology and Physiological Performance

Andrea Sabia[1]*, Ilaria Battisti[1,a], Anna Rita Trentin[1], Alessandro Alboresi[2], Tomas Morosinotto[2], Antonio Masi[1]

[1] Department of Agronomy, Food, Natural Resources, Animals, and Environment, University of Padova, Viale dell'Università 16, 35020, Legnaro, Italy

[2] Department of Biology, University of Padova, Via Ugo Bassi 58B, 35131, Padova, Italy

[a] Present address: Department of Biomedical Sciences, University of Padova, Via Ugo Bassi 58B, 35131, Padova, Italy

* Correspondence address: andrea.sabia@studenti.unipd.it

Supporting Texts

Supporting Text S1

Maize seeds were subjected to surface sterilization using 20% sodium hypochlorite for 10 minutes, followed by five consecutive rinses with sterile distilled water, each lasting 5 minutes. Seeds were hydrated in aerated sterile distilled water for 24 hours at ambient temperature, carefully transferred to trays lined with filter paper saturated with sterile water, and incubated in darkness at 25 ± 0.5 °C for 3 days to promote germination. The most uniform seedlings were selected and transplanted into tall pots containing autoclaved vermiculite, which was moistened with 100 ml of half-strength Hoagland's Nutrient Solution (NS) (refer to Supporting Table S1). Plantlets were grown in a growth chamber maintained at 24 °C and a relative air humidity range of 45-65%, with a 16-hour light photoperiod, and a photosynthetically active radiation (PAR) intensity of 170 µmol of photons m^{-2} s^{-1}. 7 days after sowing (DAS), plants were extracted from the vermiculite and transplanted to 500-ml NS polypropylene pots for 5 days to adapt to the new growing conditions. The NS was continuously oxygenated using air pumps with fine-bubble diffusers to maintain optimal root oxygenation and nutrient absorption. Plants were randomly distributed within the hydroponic system to minimize positional effects and ensure uniform exposure conditions across treatments.

Supporting Text S2

Mass spectrometry analysis was performed using a triple quadrupole instrument (TSQ Quantiva, Thermo Fisher Scientific GmbH, Bremen, Germany) interfaced with an ultra-high performance liquid chromatography (Ultimate 3000 UHPLC, Dionex, Thermo Fisher Scientific GmbH, Bremen, Germany). For each sample, 10 µl of extract were injected and separated with a flow rate of 0.4 mL min^{-1} using a C18 column (Luna® Omega PS, 1.6 µm, 50 × 2.1 mm, Phenomenex, Torrance, CA, USA) heated at 40 °C, and a delay column (Atlantis™ Premier BEH C18 AX, 5 µm, 50 × 2.1 mm, Waters, Milford, MA, USA) was introduced before the injection valve to delay the release of any possible contaminant in the mobile phase. The mobile phase was 2 mM ammonium acetate aqueous solution (A) and acetonitrile (B). The gradient started at 10% B and was maintained for 2 min, then changed to 100% B in 1 min and maintained for 5 min, then returned to 10% B in 3 min and equilibrated for 4 min. The instrument operated in selected reaction monitoring (SRM) mode, with the following parameters: negative ionization mode, spray voltage at 2500 V, ion transfer tube and vaporizer temperatures at 200 °C, collision induced dissociation (CID) argon pressure at 1.0 mTorr, sheath gas 50 (Arb), auxiliary gas 15 (Arb), and sweep gas 2 (Arb). The optimized parameters for SRM transitions are reported in the Supporting Table S3.

Supporting Tables

Supporting Table S1. Composition of the Hoagland nutrient solution (NS) used in the study. The table presents the chemical compounds and their respective concentrations in half-strength and full-strength formulations. Solutions 1-4 contain macronutrients essential for plant growth: potassium nitrate (KNO_3), calcium nitrate tetrahydrate ($Ca(NO_3)_2 \cdot 4H_2O$), magnesium sulfate heptahydrate ($MgSO_4 \cdot 7H_2O$), and monopotassium phosphate (KH_3PO_4). Solution 5 provides iron in the chelated form of $FeNaEDTA \cdot 3H_2O$. Solution 6 supplies essential micronutrients, including manganese ($MnCl_2 \cdot 4H_2O$), boron (H_3BO_3), zinc ($ZnSO_4 \cdot 7H_2O$), molybdenum ($Na_2MoO_4 \cdot 2H_2O$), and copper ($CuSO_4 \cdot 5H_2O$). Concentrations are reported in millimolar (mM) for both half- and full-strength conditions.

Solution	Compound	Half strength Concentration (mM)	Full strength Concentration (mM)
Sol. 1	KNO_3	1.5	3
Sol. 2	$Ca(NO_3)_2 \cdot 4H_2O$	1	2
Sol. 3	$MgSO_4 \cdot 7H_2O$	0.5	1
Sol. 4	KH_2PO_4	0.25	0.5
Sol. 5	$FeNaEDTA \cdot 3H_2O$	0.05	0.1
Sol. 6	$MnCl_2 \cdot 4H_2O$<br>H_3BO_3<br>$ZnSO_4 \cdot 7H_2O$<br>$Na_2MoO_4 \cdot 2H_2O$<br>$CuSO_4 \cdot 5H_2O$	0.001<br>0.0125<br>0.001<br>0.00025<br>0.00025	0.002<br>0.025<br>0.002<br>0.0005<br>0.0005

Supporting Table S2. Recovery (%) of GenX extraction by ASE, coefficient of variation CV (%), limit of detection (LOD), limit of quantification (LOQ), and correlation coefficients (R^2) of matrix-matched standard calibration curves. Recovery values indicate mean ± standard deviation.

**Parameter**	**Leaves**	**Roots**
Recovery (%)	82.0 ± 6.6	81.5 ± 4.4
CV (%)	8.0	5.4
LOD (ng g^{-1} DW)	3.5	6.6
LOQ (ng g^{-1} DW)	6.9	13.2
R^2	0.9995	0.9998

Supporting Table S3. LC-MS/MS optimized parameters for GenX detection. The first transition is the quantifier ion, and the second transition is the qualifier ion.

Molecule	Precursor m/z	Product m/z	Collision Energy (V)	RF Lens (V)
GenX	329	285<br>169	10<br>15.6	30<br>30
$^{13}C_3$-GenX	332	287<br>169	10<br>15.6	30<br>30

Supporting Table S4. Accumulation of GenX in maize leaves and roots following exposure at 0, 10, 100, and 1000 µg L^{-1}. Values are expressed as mean ± standard deviation.

GenX Accumulation in Maize Tissue (n = 4)				
**Parameter**	**0 µg L^{-1}**	**10 µg L^{-1}**	**100 µg L^{-1}**	**1000 µg L^{-1}**
[GenX] in leaves (ng g^{-1} DW)	<LOQ	160 ± 37	181 ± 37	4653 ± 1903
[GenX] in roots (ng g^{-1} DW)	<LOQ	83 ± 11	427 ± 42	3000 ± 749

Supporting Table S5. Quantitative characterization of maize root morphology traits, plant biomass, Chlorophyll and photosynthetic activity, transpiration and carboxylation rates under increasing GenX exposures (0, 10, 100, 1000 μg L^{-1}). Values represent mean ± standard deviation.

**Root Morphology (n = 12)**				
**Parameter**	**0 μg L^{-1}**	**10 μg L^{-1}**	**100 μg L^{-1}**	**1000 μg L^{-1}**
Root Surface Area (cm^2)	88 ± 10	123 ± 8	138 ± 11	105 ± 8
Root Volume (cm^3)	0.94 ± 0.18	1.25 ± 0.09	1.33 ± 0.16	1.11 ± 0.10
Root Diameter (mm)	0.40 ± 0.02	0.46 ± 0.07	0.43 ± 0.03	0.35 ± 0.03
Root Tips (nr)	1408 ± 228	3304 ± 462	2452 ± 299	1874 ± 199
**Plant Biomass (n = 6)**				
**Parameter**	**0 μg L^{-1}**	**10 μg L^{-1}**	**100 μg L^{-1}**	**1000 μg L^{-1}**
Leaves Fresh Weight (g)	3.3 ± 1.3	2.8 ± 0.6	2.8 ± 0.4	1.8 ± 0.4
Roots Fresh Weight (g)	1.9 ± 0.2	2.4 ± 0.3	2.2 ± 0.2	1.6 ± 0.3
**Chlorophyll and Photosynthetic activity (n = 12)**				
**Parameter**	**0 μg L^{-1}**	**10 μg L^{-1}**	**100 μg L^{-1}**	**1000 μg L^{-1}**
SPAD Units T0	42.6 ± 3.1	41.7 ± 4.3	41.7 ± 4.4	41.5 ± 3
SPAD Units T8	43.5 ± 3.4	47.8 ± 2.5	47.2 ± 4.0	45.1 ± 3.3
[Chl A + Chl B] μg mL^{-1}	4.10 ± 0.49	4.58 ± 0.56	4.83 ± 0.28	4.34 ± 0.46
Fv/Fm	0.779 ± 0.003	0.771 ± 0.007	0.764 ± 0.011	0.757 ± 0.012
**Transpiration and Carboxylation (n = 6)**				
**Parameter**	**0 μg L^{-1}**	**10 μg L^{-1}**	**100 μg L^{-1}**	**1000 μg L^{-1}**

Nutrient Solution Consumption (%)	38 ± 4	34 ± 3	31 ± 4	28 ± 3
Transpiration Rate (mol m^{-2} s^{-1})	0.0022 ± 0.0001	0.0018 ± 0.0003	0.0018 ± 0.0001	0.0016 ± 0.0001
CO_2 Assimilation Rate (μmol m^{-2} s^{-1})	24.8 ± 1.1	22.0 ± 2.1	22.6 ± 0.9	21.7 ± 1.8
Intercellular CO_2 (μmol mol^{-1})	142 ± 14	62 ± 20	96 ± 24	77 ± 13

Supporting Table S6. Variation in photosynthetic performance parameters in maize plants treated with GenX at 0, 10, 100, and 1000 μg L^{-1}. The table reports ϕ(II) and ϕ(I) values, measured at sequential time points and increasing light intensities. ETR(II), ETR(I), and NPQ values measured at a single light intensity of 553 μmol photons m^{-2} s^{-1} are also shown. All data are represented as mean ± standard deviation.

ϕ(II) (n = 12)					
**Time (min)**	**Light Intensity (μmol photons m^{-2} s^{-1})**	**0 μg L^{-1}**	**10 μg L^{-1}**	**100 μg L^{-1}**	**1000 μg L^{-1}**
-0.2	0	0.77 ± 0.04	0.78 ± 0.01	0.78 ± 0.01	0.72 ± 0.09
0.0	0.5	0.74 ± 0.04	0.74 ± 0.01	0.75 ± 0.01	0.68 ± 0.10
1.0	6	0.72 ± 0.04	0.73 ± 0.00	0.73 ± 0.00	0.67 ± 0.10
2.0	21	0.70 ± 0.04	0.72 ± 0.01	0.72 ± 0.01	0.66 ± 0.11
3.0	46	0.66 ± 0.04	0.70 ± 0.02	0.69 ± 0.02	0.62 ± 0.11
4.0	81	0.65 ± 0.04	0.66 ± 0.02	0.64 ± 0.03	0.58 ± 0.11
5.0	171	0.57 ± 0.04	0.57 ± 0.02	0.55 ± 0.04	0.50 ± 0.10
7.0	279	0.49 ± 0.04	0.44 ± 0.06	0.39 ± 0.07	0.40 ± 0.10
8.0	336	0.45 ± 0.04	0.37 ± 0.06	0.32 ± 0.07	0.34 ± 0.09
9.0	427	0.39 ± 0.04	0.29 ± 0.05	0.25 ± 0.06	0.27 ± 0.07
10.0	553	0.32 ± 0.03	0.23 ± 0.04	0.19 ± 0.04	0.22 ± 0.06
11.0	684	0.26 ± 0.02	0.18 ± 0.03	0.15 ± 0.03	0.17 ± 0.05
12.0	856	0.21 ± 0.02	0.15 ± 0.02	0.13 ± 0.03	0.14 ± 0.04
13.0	1034	0.18 ± 0.03	0.12 ± 0.02	0.10 ± 0.02	0.11 ± 0.03
14.0	1283	0.14 ± 0.01	0.10 ± 0.01	0.08 ± 0.02	0.09 ± 0.02

15.0	1619	0.11 ± 0.01	0.08 ± 0.01	0.07 ± 0.01	0.07 ± 0.02
16.0	2028	0.09 ± 0.01	0.06 ± 0.01	0.05 ± 0.01	0.06 ± 0.01
18.0	0	0.65 ± 0.05	0.66 ± 0.02	0.66 ± 0.01	0.57 ± 0.13
19.0	0	0.69 ± 0.05	0.71 ± 0.01	0.71 ± 0.01	0.62 ± 0.14
20.0	0	0.71 ± 0.05	0.72 ± 0.01	0.72 ± 0.01	0.63 ± 0.14

ϕ(I) (n = 12)

**Time (min)**	**Light Intensity (µmol photons m^{-2} s^{-1})**	**0 µg L^{-1}**	**10 µg L^{-1}**	**100 µg L^{-1}**	**1000 µg L^{-1}**
-0.2	0	0.73 ± 0.19	0.66 ± 0.09	0.65 ± 0.13	0.62 ± 0.13
0.0	0.5	0.41 ± 0.08	0.41 ± 0.05	0.42 ± 0.07	0.42 ± 0.04
1.0	6	0.57 ± 0.14	0.49 ± 0.03	0.51 ± 0.03	0.56 ± 0.19
2.0	21	0.56 ± 0.14	0.51 ± 0.04	0.50 ± 0.03	0.55 ± 0.16
3.0	46	0.57 ± 0.11	0.51 ± 0.05	0.50 ± 0.03	0.56 ± 0.14
4.0	81	0.60 ± 0.11	0.52 ± 0.06	0.50 ± 0.03	0.57 ± 0.11
5.0	171	0.64 ± 0.08	0.59 ± 0.04	0.59 ± 0.04	0.61 ± 0.06
7.0	279	0.64 ± 0.04	0.62 ± 0.04	0.60 ± 0.04	0.59 ± 0.05
8.0	336	0.62 ± 0.05	0.58 ± 0.05	0.56 ± 0.05	0.56 ± 0.07
9.0	427	0.58 ± 0.04	0.51 ± 0.05	0.50 ± 0.04	0.51 ± 0.07
10.0	553	0.52 ± 0.04	0.44 ± 0.05	0.44 ± 0.04	0.44 ± 0.06
11.0	684	0.47 ± 0.04	0.39 ± 0.04	0.38 ± 0.04	0.39 ± 0.05
12.0	856	0.40 ± 0.04	0.34 ± 0.03	0.33 ± 0.03	0.33 ± 0.03
13.0	1034	0.37 ± 0.04	0.29 ± 0.03	0.29 ± 0.03	0.29 ± 0.03

14.0	1283	0.31 ± 0.03	0.25 ± 0.02	0.25 ± 0.02	0.25 ± 0.02
15.0	1619	0.26 ± 0.02	0.21 ± 0.02	0.21 ± 0.02	0.21 ± 0.02
16.0	2028	0.22 ± 0.02	0.18 ± 0.01	0.17 ± 0.01	0.18 ± 0.01
18.0	0	0.61 ± 0.08	0.56 ± 0.03	0.54 ± 0.04	0.60 ± 0.12
19.0	0	0.60 ± 0.08	0.55 ± 0.03	0.53 ± 0.03	0.59 ± 0.13
20.0	0	0.60 ± 0.08	0.56 ± 0.03	0.53 ± 0.03	0.60 ± 0.14

ETR(II) (n = 6)

Time (min)	Light Intensity (μmol photons m^{-2} s^{-1})	0 μg L^{-1}	10 μg L^{-1}	100 μg L^{-1}	1000 μg L^{-1}
-0.3	0	0.0	0.0	0.0	0.0
0	0	0.0	0.0	0.0	0.0
0.5	553	4.3 ± 1.5	3.3 ± 1.3	2.9 ± 1.2	5.3 ± 1.9
1	553	26.0 ± 5.3	24.3 ± 9.3	24.7 ± 7.6	30.0 ± 9.2
1.5	553	36.6 ± 8.8	36.2 ± 5.0	36.5 ± 4.4	41.7 ± 9.2
2	553	50.5 ± 7.4	40.7 ± 6.0	40.5 ± 4.6	43.4 ± 8.7
2.5	553	54.9 ± 8.8	41.5 ± 5.9	39.5 ± 5.8	42.6 ± 8.2
3	553	57.2 ± 9.2	42.3 ± 5.7	39.2 ± 5.0	42.9 ± 8.5
3.5	553	58.9 ± 8.8	42.3 ± 5.1	39.4 ± 4.8	43.2 ± 8.8
4	553	60.5 ± 8.2	42.6 ± 4.9	40.0 ± 4.7	44.1 ± 8.8
4.5	553	62.3 ± 7.2	43.0 ± 5.1	40.7 ± 5.0	45.1 ± 9.1
5	553	63.6 ± 6.6	43.5 ± 5.1	41.5 ± 5.2	45.5 ± 8.5
5.5	553	64.4 ± 5.9	44.0 ± 5.2	42.4 ± 5.6	45.8 ± 8.6

6	553	65.5 ± 5.3	44.3 ± 5.3	43.2 ± 6.1	46.3 ± 8.5
6.5	553	66.4 ± 4.8	44.7 ± 5.4	43.8 ± 6.7	46.9 ± 8.4
7	553	67.3 ± 4.5	45.1 ± 5.3	44.5 ± 7.3	47.3 ± 8.1
7.5	553	68.3 ± 4.4	45.8 ± 5.8	45.2 ± 7.9	48.4 ± 7.6
8	553	69.0 ± 4.3	46.0 ± 5.4	45.9 ± 8.8	48.5 ± 8.0
8.5	0	0.0	0.0	0.0	0.0
9	0	0.0	0.0	0.0	0.0
10	0	0.0	0.0	0.0	0.0
11	0	0.0	0.0	0.0	0.0

ETR(I) (n = 6)

**Time (min)**	**Light Intensity (μmol photons m^{-2} s^{-1})**	**0 μg L^{-1}**	**10 μg L^{-1}**	**100 μg L^{-1}**	**1000 μg L^{-1}**
-0.3	0	0.0	0.0	0.0	0.0
0	0	0.0	0.0	0.0	0.0
0.5	553	7.0 ± 1.0	5.6 ± 1.2	5.4 ± 1.1	6.8 ± 3.1
1	553	91.1 ± 21.0	72.9 ± 9.1	71.7 ± 4.1	75.5 ± 6.2
1.5	553	118.8 ± 25.0	79.4 ± 6.1	81.5 ± 4.4	86.2 ± 6.5
2	553	114.1 ± 19.4	76.3 ± 4.1	76.8 ± 4.1	78.3 ± 6.4
2.5	553	103.0 ± 18.1	72.8 ± 3.6	75.3 ± 2.4	73.4 ± 4.1
3	553	99.5 ± 15.8	73.2 ± 4.0	75.3 ± 8.2	72.3 ± 4.4
3.5	553	99.7 ± 14.2	73.6 ± 6.8	76.0 ± 7.6	72.6 ± 4.5
4	553	100.8 ± 12.4	73.8 ± 7.1	76.5 ± 7.5	72.4 ± 13.7

4.5	553	102.8 ± 11.7	74.0 ± 7.5	76.1 ± 6.6	73.5 ± 15.3
5	553	103.0 ± 11.8	74.0 ± 6.8	76.1 ± 6.4	72.9 ± 13.3
5.5	553	104.2 ± 11.5	74.1 ± 6.6	76.6 ± 7.0	73.0 ± 13.3
6	553	105.5 ± 10.5	73.7 ± 6.5	76.3 ± 6.4	73.4 ± 13.3
6.5	553	106.6 ± 10.3	74.2 ± 7.1	77.1 ± 7.1	73.8 ± 13.4
7	553	107.8 ± 10.3	74.3 ± 6.7	76.7 ± 6.8	74.1 ± 12.8
7.5	553	108.8 ± 9.5	74.1 ± 7.1	76.5 ± 7.3	74.2 ± 13.2
8	553	110.0 ± 8.9	74.2 ± 6.7	76.3 ± 6.8	74.7 ± 13.3
8.5	0	0.0	0.0	0.0	0.0
9	0	0.0	0.0	0.0	0.0
10	0	0.0	0.0	0.0	0.0
11	0	0.0	0.0	0.0	0.0

NPQ (n = 6)

**Time (min)**	**Light Intensity (μmol photons m^{-2} s^{-1})**	**0 μg L^{-1}**	**10 μg L^{-1}**	**100 μg L^{-1}**	**1000 μg L^{-1}**
-0.3	0	0.00	0.00	0.00	0.00
0	0	0.03 ± 0.00	0.03 ± 0.00	0.03 ± 0.00	0.03 ± 0.01
0.5	553	0.01 ± 0.01	0.01 ± 0.01	0.01 ± 0.01	0.15 ± 0.21
1	553	0.18 ± 0.08	0.22 ± 0.13	0.23 ± 0.06	0.23 ± 0.05
1.5	553	0.21 ± 0.09	0.27 ± 0.16	0.25 ± 0.08	0.27 ± 0.07
2	553	0.35 ± 0.08	0.43 ± 0.20	0.40 ± 0.12	0.43 ± 0.10
2.5	553	0.52 ± 0.10	0.63 ± 0.19	0.59 ± 0.13	0.59 ± 0.06

3	553	0.60 ± 0.12	0.76 ± 0.19	0.70 ± 0.14	0.67 ± 0.08
3.5	553	0.66 ± 0.13	0.85 ± 0.20	0.78 ± 0.15	0.75 ± 0.10
4	553	0.72 ± 0.13	0.92 ± 0.22	0.85 ± 0.14	0.83 ± 0.11
4.5	553	0.76 ± 0.13	0.98 ± 0.23	0.92 ± 0.13	0.89 ± 0.09
5	553	0.79 ± 0.12	1.04 ± 0.23	0.98 ± 0.13	0.96 ± 0.08
5.5	553	0.81 ± 0.11	1.08 ± 0.23	1.03 ± 0.13	1.01 ± 0.07
6	553	0.83 ± 0.10	1.12 ± 0.22	1.07 ± 0.13	1.05 ± 0.06
6.5	553	0.84 ± 0.09	1.16 ± 0.22	1.10 ± 0.14	1.08 ± 0.06
7	553	0.84 ± 0.09	1.18 ± 0.21	1.13 ± 0.14	1.11 ± 0.06
7.5	553	0.85 ± 0.09	1.21 ± 0.21	1.15 ± 0.15	1.13 ± 0.06
8	553	0.85 ± 0.09	1.23 ± 0.20	1.17 ± 0.15	1.15 ± 0.07
8.5	0	0.85 ± 0.10	1.25 ± 0.19	1.19 ± 0.15	1.10 ± 0.22
9	0	0.42 ± 0.06	0.61 ± 0.08	0.60 ± 0.10	0.49 ± 0.09
10	0	0.22 ± 0.05	0.28 ± 0.01	0.28 ± 0.03	0.27 ± 0.04
11	0	0.16 ± 0.05	0.18 ± 0.01	0.19 ± 0.02	0.20 ± 0.04

Supporting Table S7. Relative variation (%) in photosynthetic protein abundance in leaves following GenX exposure. The table reports the percentage change in abundance of key thylakoid proteins involved in photosynthesis and energy dissipation, such as γ-ATPase, PSAA, D2, LHCII, and PsbS, after treatment with GenX at 10, 100, and 1000 μg L^{-1}. Protein levels were quantified by immunoblot analysis and normalized to control samples. 'NS' indicates samples where the difference with the control was not significant. All data are represented as mean ± standard deviation.

Protein	10 μg L^{-1}	100 μg L^{-1}	1000 μg L^{-1}
γ-ATPase	-86 ± 2	-87 ± 2	-80 ± 12
PSAA	NS	NS	NS
D2	NS	NS	NS
LHCII	+137 ± 11	+118 ± 3	+101 ± 20
PsbS	+45 ± 17	+49 ± 8	+36 ± 10

Supporting Figures

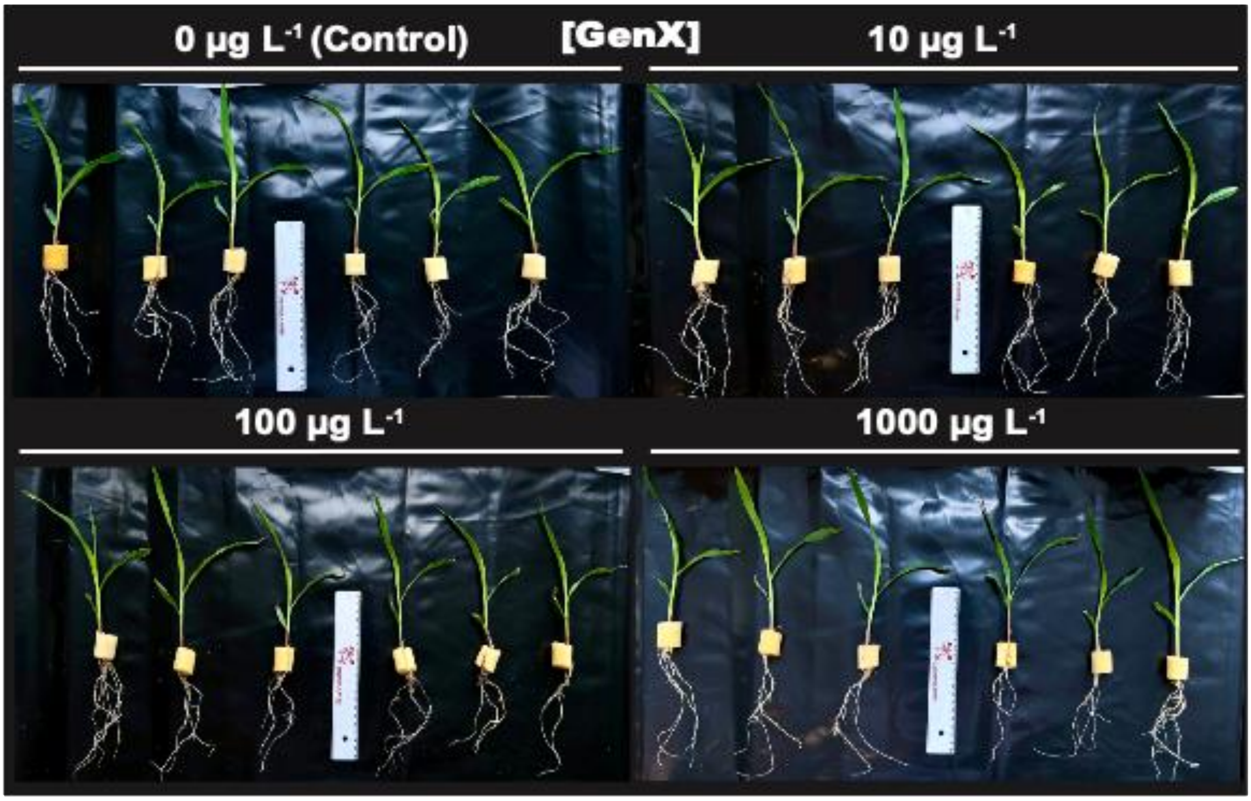

Supporting Figure S1. Plant images acquired at 12 DAS. Plants were divided as equally as possible by the size of the aerial and root portions. This growth stage represents the day of the start of treatment (DAT0) with GenX for control (0 µg L^{-1}) and concentrations of 10, 100, and 1000 µg L^{-1}. Each group consists of six biological replicates (n = 6).

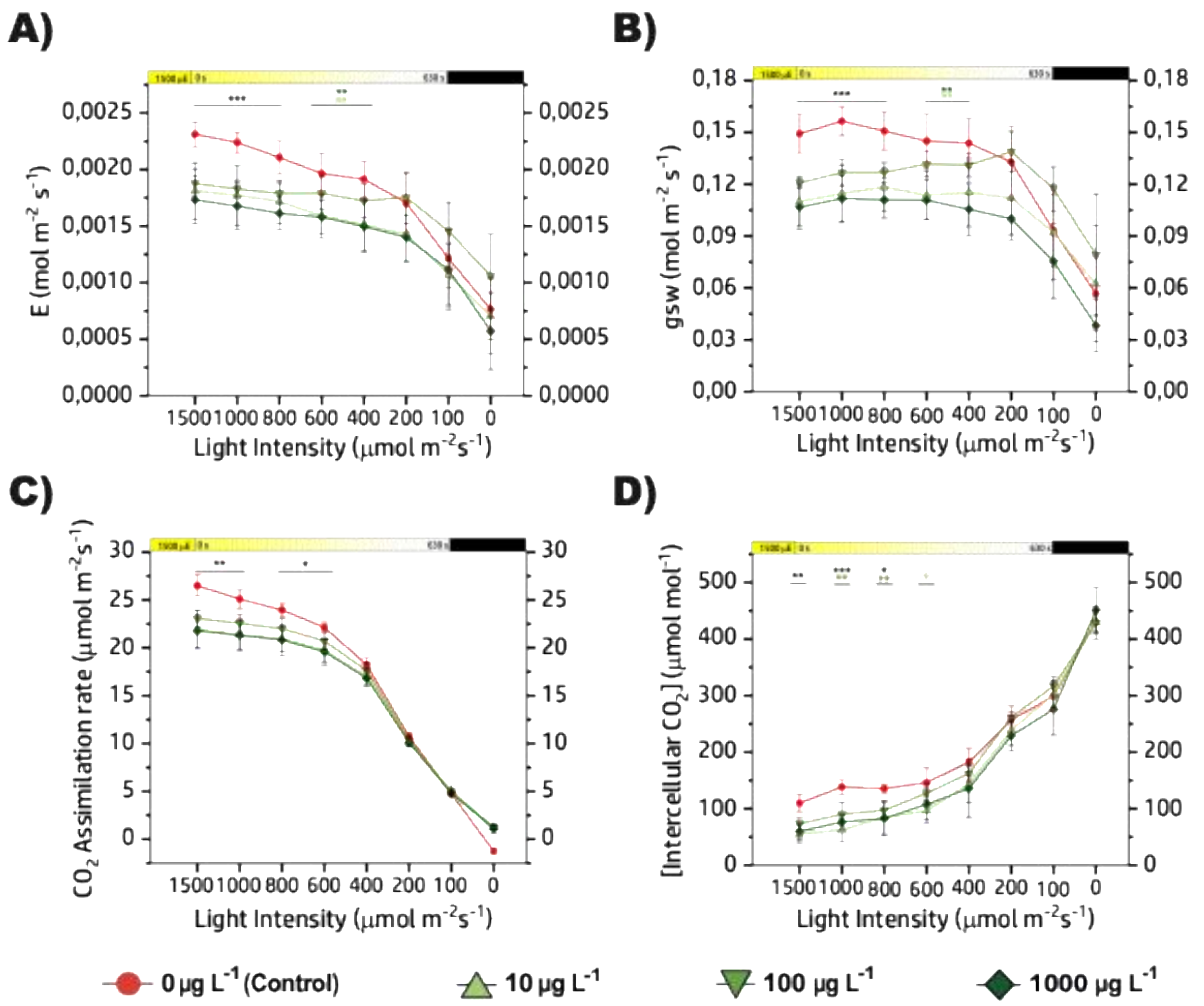

Supporting Figure S2. Gas exchange parameters were monitored during light exposure for control plants (0 µg L^{-1}) and treated plants (10, 100 and 1000 µg L^{-1}) and are represent as follows: (A) transpiration rate (mol m^{-2} s^{-1}), (B) stomatal conductance (mol m^{-2} s^{-1}), (C) CO_2 assimilation rate (µmol m^{-2} s^{-1}), (D) intercellular CO_2 (µmol mol^{-1}). After light adaptation of 15 minutes at 1500 µmol photons m^{-2} s^{-1} (1st yellow bar), plants were exposed to a decreasing light curve kinetics (from 1500 µmol m^{-2} s^{-1} to 100 µmol m^{-2} s^{-1}, white and yellow bars) for 10.5 mins, followed by a rapid recovery in the dark for 1 min (black bar). Statistical significance is indicated by black asterisks (* $p < 0.05$, ** $p < 0.01$, *** $p < 0.001$, **** $p < 0.0001$, two-way ANOVA). Data are shown in red for control (0 µg L^{-1}), whereas plants exposed to GenX (10, 100, and 1000 µg L^{-1}) are colored in shades of green.
